# The effect of CA125 on metastasis of ovarian cancer: old marker new function

**DOI:** 10.18632/oncotarget.18388

**Published:** 2017-06-07

**Authors:** Qin Yuan, Jiayin Song, Weiwei Yang, Hongyan Wang, Qianyu Huo, Jie Yang, Xiaoxu Yu, Yunde Liu, Chen Xu, Huijing Bao

**Affiliations:** ^1^ School of Laboratory Science, Tianjin Medical University, Tianjin, China; ^2^ The Department of Clinical Laboratory, National Clinical Research Center for Cancer, Tianjin Medical University Cancer Institute and Hospital, Tianjin, China; ^3^ The Department of Laboratory Science, Tianjin Central Hospital of Gynecology Obstetrics, Tianjin, China; ^4^ The Department of Laboratory Science, Tianjin Fourth Central Hospital, Tianjin, China

**Keywords:** ovarian cancer, metastasis, CA125, Wnt pathway, cut-off value

## Abstract

CA125 has been used extensively to screen for neoplasms, especially in ovarian cancer. The serum CA125 level can be used as a better prognosis evaluation and it may dynamic monitoring the disease progression. We explored the effect of CA125 on ovarian cancer cell migration and its underlying mechanism. Transwell assays showed that exposure to 0.2 μg/ml or 0.4 μg/ml CA125 for 48 h increased migration of A2780 and OVCAR-3 ovarian cancer cells. This effect of CA125 was blocked addition of 200 ng/ml DKK-1, a Wnt pathway inhibitor. Conversely, addition of CA125 reversed the inhibitory effect of Wnt inhibition in A2780 cells pretreated with DKK-1. Examination of CA125 levels in serum from 97 ovarian cancer patients revealed no relationship between a patient's age or CA125 level currently used clinically for ovarian cancer diagnosis and metastasis. However, using receiver operating characteristic (ROC) curves, we identified a new cut-off value for the serum CA125 concentration (82.9 U/ml) that is predictive of metastasis. The area under the curve is 0.632. This new cut-off value has the potential to serve as a clinically useful indicator of metastasis in ovarian cancer patients.

## INTRODUCTION

In 2012, ovarian cancer was as seventh leading tumor in women, but the leading cause of cancer-related death due to its late diagnosis [1, 2]. Many reports have focused on the diagnostic value of CA125 in ovarian cancer, and have advocated for increased use of serum CA125 in primary care as means of increasing detection of ovarian cancer [3, 4]. CA125 is member of the tethered human mucus (MUC) family of large, heavily glycosylated transmembrane proteins that have a diverse range of functions [5]. The extracellular region of MUC16, which is over-expressed in epithelial ovarian carcinoma, is cleaved from the cell surface and circulates in the blood. Known as CA125, this protein is a well-established ovarian cancer marker used for clinical diagnosis [6]. CA125 levels higher than 35 U/mL are considered abnormal and are associated with 90% of ovarian carcinomas, and are strongly associated with a poor prognosis [7]. Serum CA125 levels are routinely monitored in patients with ovarian cancer, and an increase from an individualized nadir concentration is a prognostic indicator of cancer recurrence. More than that, CA125 levels are useful indicators of the response to chemotherapy and disease relapse and progression [10, 11].

In the present study, we examined the relationship between serum CA125 levels and ovarian cancer metastasis, and evaluated the mechanism underlying that relationship. We also discuss whether serum CA125 levels could serve as a clinically useful predictor of metastasis.

## RESULTS

### CA125 has no remarkable effect on ovarian cancer cell proliferation

**Figure 1:**
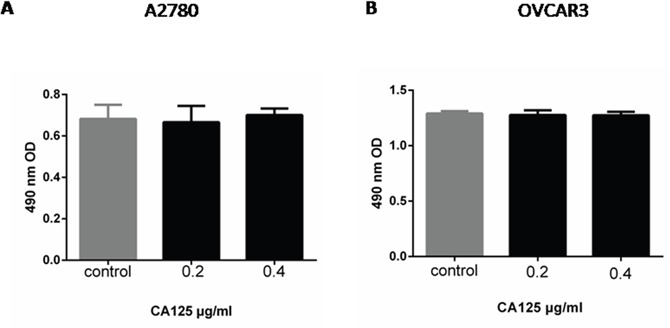
CA125 has little effect on ovarian cancer cell proliferation MTT assays were performed after incubating A2780 **(A)** and OVCAR-3 **(B)** cells for 48 h in the presence of 0.2 μg/ml or 0.4 μg/ml CA125. Data were analyzed using one-way ANOVA (n = 3).

MTT assays were used to evaluate the proliferation of A2780 or OVCAR-3 ovarian cancer cells. We observed no significant effect of CA125 on cell proliferation. It is obvious that the proliferative capacity of A2780 and OVCAR-3 cells was unaffected by incubation with 0.2 μg/ml or 0.4 μg/ml CA125 (Figure 1A and 1B).

### CA125 promotes ovarian cancer cell migration

**Figure 2:**
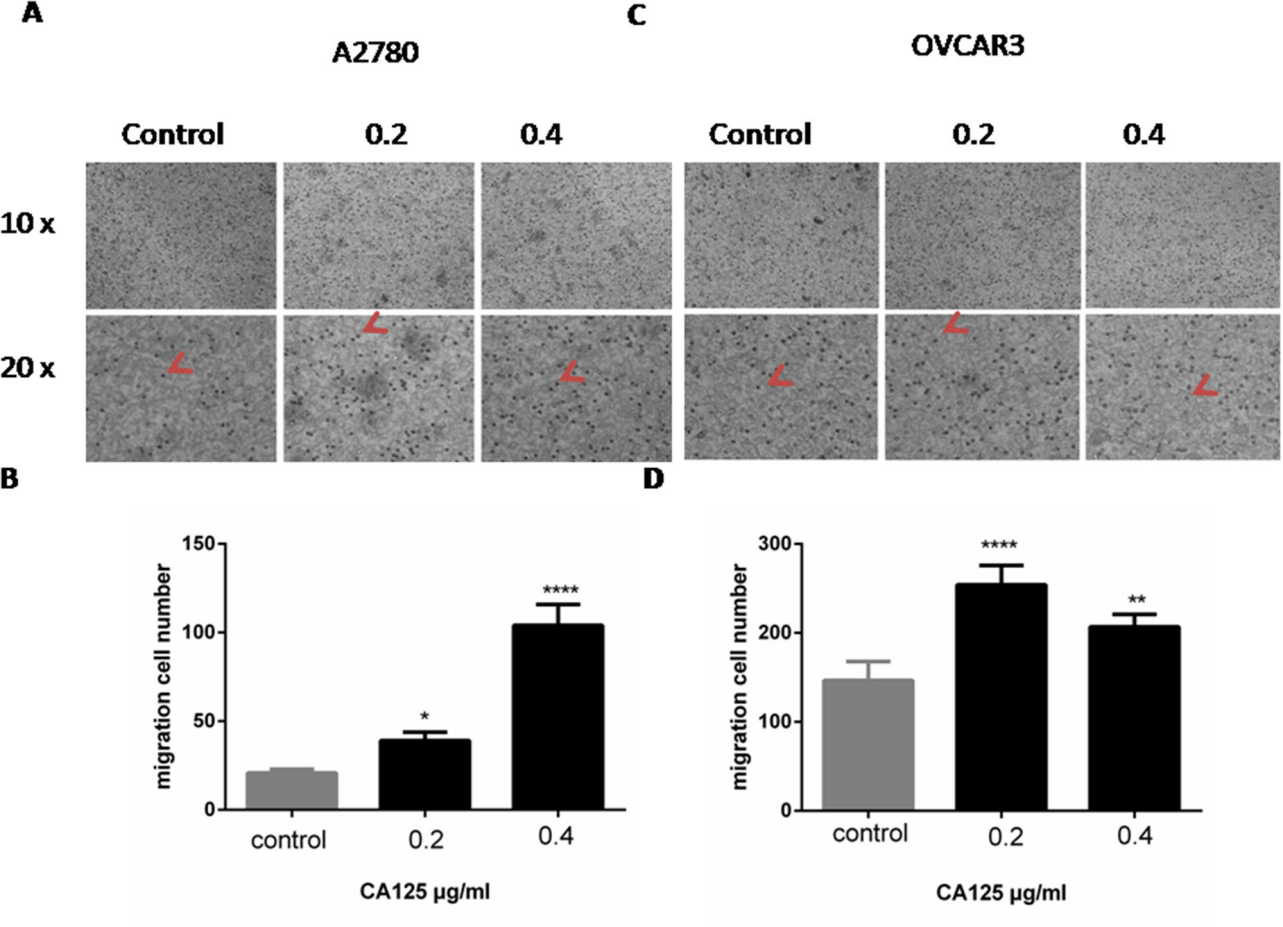
CA125 increases ovarian cancer cell migration Transwell migration assays were performed to assess migration of A2780 **(A, B)** and OVCAR-3 **(C, D)** cells exposed to 0.2 μg/ml or 0.4 μg/ml CA125 for 48 h. The cell number was counted by counting black spot in the high magnification image (red arrow pointed). All the data were normally distributed, and one-way ANOVA was used to analyze them. (n=3). *P < 0.05; **P < 0.01.

Transwell migration assays showed that incubation for 48 h in medium containing 0.2 μg/ml or 0.4 μg/ml CA125 significantly increased the numbers of migrated A2780 cells (Figure 2A), and that the effect was concentration-dependent (Figure 2B). CA125 (0.2 μg/ml or 0.4 μg/ml) had a similar effect on OVCAR-3 cells (Figure 2C), though the elicited response was not concentration-dependent. When the OVCAR-3 cells were incubated with CA125, similar significant increases in the number of migrated cells were detected at both CA125 concentrations (Figure 2D).

### CA125-induced cell migration may depend on the Wnt signaling pathway

**Figure 3:**
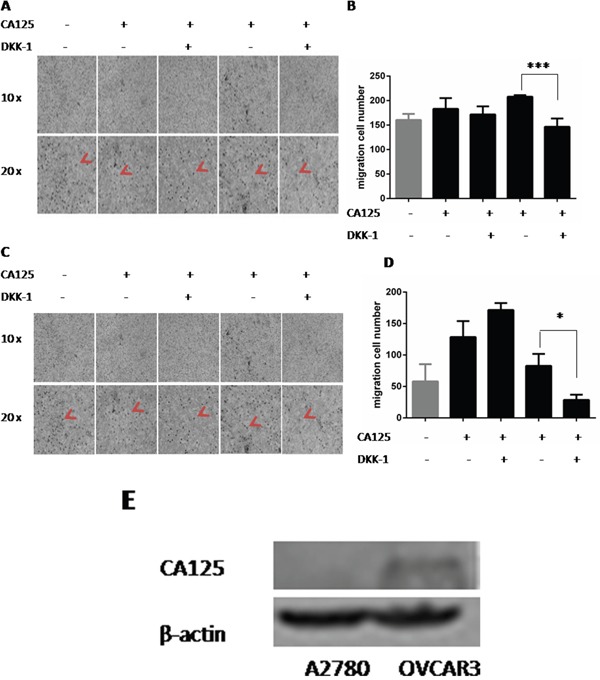
CA125 increases cell migration via the Wnt signaling pathway Transwell migration assays were performed to assess the effect the DKK-1 (200 ng/ml), a Wnt pathway inhibitor, on migration of A2780 **(A, B)** and OVCAR-3 **(C, D)** cells exposed to 0.2 μg/ml or 0.4 μg/ml CA125 for 48 h. The cell number was counted by counting black spot in the high magnification image (red arrow pointed). **(E)** Western blot analysis of CA125 levels in A2780 and 0VCAR3 cells. The data were normally distributed, and they were analyzed using one-way ANOVA (n=3). *P < 0.05; **P < 0.01.

The Wnt signaling pathway is involved in various malignant tumors, including ovarian cancer [6, 12–14]. We therefore assessed the contribution made by Wnt signaling to CA125-induced ovarian cancer cell migration. We discovered that the capacity of CA125 to increase A2780 cell migration was reduced when the cells were incubated for 48 h with DKK-1 (200 ng/ml), a Wnt signaling inhibitor (Figure 3A). The numbers of cells migrating in the presence of 0.4 μg/ml CA125 were significant decreasing in the presence of 200 ng/ml DKK-1 (Figure 3B).

A somewhat different response to DKK-1 was observed with OVCAR- 3 cells. Incubating the cells with CA125 (0.2 μg/ml) plus DKK-1 (200 ng/ml) actually increased the number of migrated cells, as compared to migration in response to the CA125 alone. By contrast, as with A2780 cells, DKK-1 exerted an inhibitory effect on the migration of OVCAR- 3 cells incubated with the higher dose of CA125 (0.4 μg/ml) (Figure 3C and 3D).

We suggest their different genetic backgrounds are responsible for the different responses of the two cell lines to CA125 and DKK-1. Whereas A2780 is considered a CA125^−^ cell line, OVCAR-3 is a CA125^+^ cell line. Using western blotting, we confirmed that OVCAR-3 cells express higher levels of CA125 than A2780 cells (Figure 3E). In both cell types, however, Wnt pathway appears to play an important role in mediating the positive effect of CA125 on ovarian cancer cell migration.

### CA125 reverses the effect Wnt pathway inhibition on cancer cell migration

**Figure 4:**
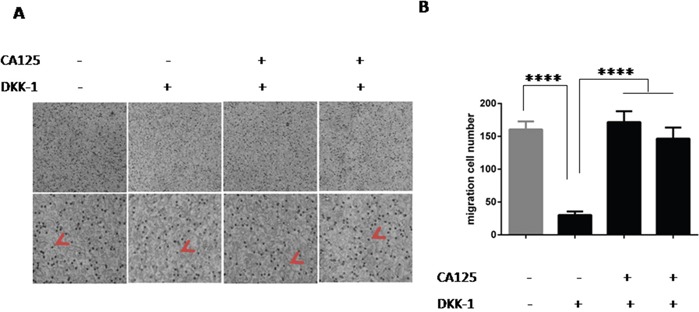
CA125 reverses the effect Wnt pathway inhibition on cancer cell migration **(A)** Transwell migration assays were performed to assess the ability of CA125 (0.2 μg/ml or 0.4 μg/ml) to reverse DKK-1 (200 ng/ml)-induced suppression of migration. After incubation, the photos were taken with an inverted phase contrast microscope. **(B)** High magnification image of the cells used for the assay. The cell number was counted by counting black spot in the high magnification image (red arrow pointed). The data were normally distributed, and they were analyzed using one-way ANOVA (n=3). *P < 0.05; **P < 0.01; ***P < 0.001, **** P < 0.0001

To further examination of the relationship between CA125, the Wnt pathway, and ovarian cancer cell migration, we next performed a rescue experiment. Adding DKK-1 (200 ng/ml) to the culture medium elicited a marked decline in A2780 cell migration. Notably, subsequent addition of the CA125 (0.2 μg/ml or 0.4 μg/ml) to the same medium reversed the decline in cell migration (Figure 4A and 4B). Apparently, CA125 can reverse the suppressive effect of Wnt pathway inhibition on cancer cell migration.

### Serum CA125 levels may indicate ovarian cancer metastasis

**Figure 5:**
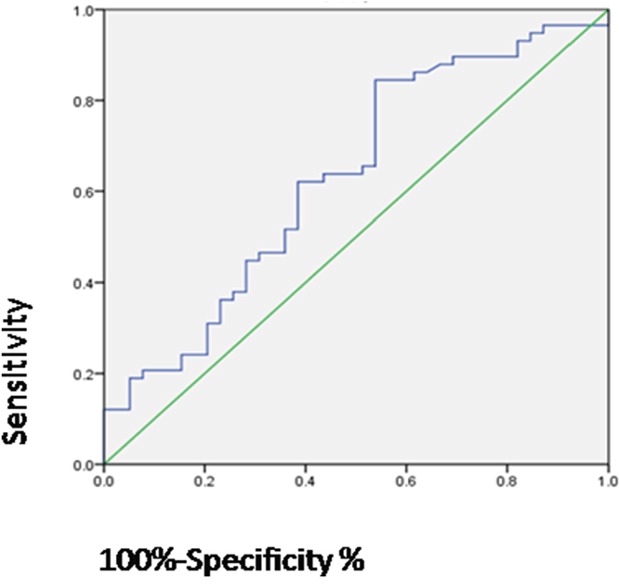
Serum CA125 level predictive of metastasis in ovarian cancer Graph showing the ROC curve used to determine the cut-off serum CA125 level for prediction of ovarian cancer metastasis. The area under the ROC curve = 0.632, p-value is 0.043.

Our observation that CA125 increases A2780 and OVCAR-3 cell migration suggests that CA125 may play a key role in ovarian cancer metastasis and that its level in serum may be predictive of metastasis. To test that idea, we collected the clinical data for 97 ovarian cancer patients (Supplementary Table 1) from the Laboratory Science Department of Tianjin Cancer Hospital of Tianjin Medical University. The ethics committee of the Tianjin Medical University Cancer Hospital approved the use of human samples for this study, and each patient signed an informed consent form for participation. Thirty-three of the patients were under 50 years old, while 64 were patients are more than 50 years old. Among the 97 patients, 58 showed tumor metastasis. The median values for serum CA125 in metastatic and non-metastatic patients were 187.9 and 408.25, respectively. The clinically diagnostic level of CA125 in ovarian cancer is 35 U/ml [15–17]. Analysis using McNemar's test revealed there is no significant correlation between 35 U/ml or age and ovarian cancer metastasis (Table 1). After dividing the data into metastasis and no-metastasis groups, a receiver operating characteristic (ROC) curve was used to determine the CA125 concentration most predictive of ovarian cancer metastasis. According to the ROC results, a new cut-off value of 82.9 U/ml was identified, and the area under the curve was 0.632 (Figure 5).

**Table 1:** Factors associated with ovarian cancer metastasis

	Number	Metastasis		P
		Y	N	
**CA125 Level (U/ml)**				
<35	12	4	8	0.203
≥35	85	54	31	
**Age**				
<50	33	19	14	0.032
≥50	64	39	25	

## DISCUSSION

Ovarian cancer is the leading cause of death among gynecological cancers primarily due to its late detection and peritoneal dissemination [18–22]. Patients with ovarian cancer are often diagnosed at an advanced stage, after tumors have spread into the peritoneal cavity. And although the standard therapy involves aggressive surgery followed by platinum/taxane-based chemotherapy, the majority of patients experience relapse with chemoresistant disease [23]. It is therefore important to identify factors associated with ovarian cancer metastasis or recurrence. There is reportedly a strong positive relationship between recurrence and serum CA125 levels after surgery. In our study, we focused on the role of serum CA125 in ovarian cancer metastasis and established a new CA125 cut-off concentration to use for predication of ovarian cancer metastasis.

We found that ovarian cancer cells proliferation was not greatly affected by CA125, but migration was strongly increasing by two concentrations of CA125. It appears the stimulatory effect of CA125 on cell migration is mediated via the Wnt signaling pathway: migration was inhibited by the Wnt antagonist DKK-1, and DKK-1-mediated suppression of cell migration was revised by CA125. Interesting, we observed small differences in the responses of A2780 and OVCAR-3 cells to CA125 and DKK-1. We think this reflects the fact that A2780 cells are CA125^−^, while OVCAR-3 is CA125^+^ and secretes CA125 spontaneously. In both cell types, however, the Wnt pathway appears to play will important role in the CA125 effect on ovarian cancer migration.

To determine whether our *in vitro* observations have a potential clinical application, serum CA125 levels from ovarian cancer 97 patients with were collected and analyzed. We found that metastasis has no relationship with a patient's age or the serum CA125 level currently used clinically for diagnosis (35 U/ml). However, ROC curve analysis suggested a level of 82.9 U/ml was predictive of ovarian cancer tumor metastasis. This new cut-off value for serum CA125 level could potentially serve as an effective indicator of ovarian cancer metastasis.

In summary, we have shown that CA125 stimulates ovarian cancer cell migration, and that this effect is mediated via the Wnt signaling pathway. More than that, we have established a new cut-off value for serum CA125 (82.9 U/ml) that may be predictive of metastasis in patients with ovarian cancer.

## MATERIALS AND METHODS

### Cell lines

The A2780 and OVCAR-3 ovarian cancer cell lines were purchased from the American Type Culture Collection (ATCC) and cultured in our laboratory. A2780 cells were cultured in RPMI 1640 medium supplemented with 10% fetal bovine serum (FBS). OVCAR-3 cells were cultured in 1640 RPMI supplemented with 10% FBS and 1% insulin (all from Sigma Chemical, St. Louis, MO). Both lines were incubated at 37°C under 5% CO_2_.

### Cell proliferation assay

The cell proliferation was assayed was performed using MTT assays according to the manufacturer's instruction and as previously described. Briefly, 5×10^4^ cells per well were incubated for 48 h in medium with or without CA125 (0.2 μg/ml or 0.4 μg/ml). For colorimetric analysis, absorbance was read at 490 nm using a microplate reader (Biotek, USA). Each experiment was repeated at least 3 times.

### Transwell migration assay

A2780 or OVCAR3 cells were seeded to density of 5×10^4^ cells per well in transwell inserts (polycarbonate filters with 8 μm pores, Costor) filled with medium containing 0.2 μg/ml or 0.4 μg/ml CA125 and/or 200 ng/ml DKK-1(the classical Wnt pathway inhibitor, from Peprotech). The bottom chamber contained 600 μl of medium. After incubation for 48 h, cells remaining in the upper chamber were gently removed using a cotton swap. Cells that migrated to the lower surface of the filter membrane were fixed with anhydrous methanol and stained with 0.1% crystal violet. The filters were then air-dried, and photos were taken using an inverted phase contrast microscope (Nikon ECLIPSE Ti-U).

### Western blot

A standard protocol was used for western blotting. Cell lysis was carried out as previously described [6, 25]. Protein concentrations were determined using BCA assays. The primary antibodies used were goat anti-CA125 (dilution, 1:1000; Abcam) and rabbit anti-β-actin (dilution, 1:1000; Abcam). The secondary antibodies were mouse anti-goat IgG (dilution, 1:1000; Affinity) and goat anti-rabbit IgG (dilution, 1:1000; Affinity). To detect differences in CA125 levels, proteins in total cell lysates were analyzed on separate gels.

### Statistical analysis

SPSS software was used for statistical analyses. Results are expressed as the mean ± SD or mean ± SEM. The significance of differences of variables between two groups was determined using the two-tailed unpaired Student's t tests. One-way ANOVA was applied to compare variables among three samples groups. Values of P < 0.05 were considered significant.

## SUPPLEMENTARY INFORMATION TABLE





## References

[1] Davidson B, Tropé CG (2014). Ovarian cancer: diagnostic, biological and prognostic aspects. Womens Health (Lond).

[2] Garcia-Perez J, Lope V, Lopez-Abente G, Gonzalez-Sanchez M, Fernandez-Navarro P (2015). Ovarian cancer mortality and industrial pollution. Environ Pollut.

[3] Moss EL, Moran A, Reynolds TM, Stokes-Lampard H (2013). Views of general practitioners on the role of CA125 in primary care to diagnose ovarian cancer. BMC Womens Health.

[4] Pelissier A, Bonneau C, Chereau E, de La Motte Rouge T, Fourchotte V, Darai E, Rouzier R (2014). Ca125 kinetic parameters predict optimal cytoreduction in patients with advanced epithelial ovarian cancer treated with neoadjuvant chemotherapy. Gynecol Oncol.

[5] Williams KA, Terry KL, Tworoger SS, Vitonis AF, Titus LJ, Cramer DW (2014). Polymorphisms of Muc16 (Ca125) and Muc1 (Ca15.3) in relation to ovarian cancer risk and survival. PLoS One.

[6] Liu Q, Cheng Z, Luo L, Yang Y, Zhang Z, Ma H, Chen T, Huang X, Lin SY, Jin M, Li Q, Li X (2016). C-terminus of MUC16 activates Wnt signaling pathway through its interaction with β-catenin to promotetumorigenesis and metastasis. Oncotarget.

[7] Das S, Majhi PD, Al-Mugotir MH, Rachagani S, Sorgen P, Batra SK (2015). Membrane proximal ectodomain cleavage of Muc16 occurs in the acidifying golgi/post-golgi compartments. Sci Rep.

[8] Felder M, Kapur A, Gonzalez-Bosquet J, Horibata S, Heintz J, Albrecht R, Fass L, Kaur J, Hu K, Shojaei H, Whelan RJ, Patankar MS (2014). MUC16 (CA125): tumor biomarker to cancer therapy, a work in progress. Mol Cancer.

[9] Danilos J, Michal Kwasniewski W, Mazurek D, Bednarek W, Kotarski J (2015). Meigs' syndrome with elevated Ca-125 and He-4: a case of luteinized fibrothecoma. Prz Menopauzalny.

[10] Morales-Vasquez F, Pedernera E, Reynaga-Obregon J, Lopez-Basave HN, Gomora MJ, Carlon E, Cardenas S, Silva-Ayala S, Almaraz M, Mendez C (2016). High Levels of pretreatment Ca125 are associated to improved survival in high grade serous ovarian carcinoma. J Ovarian Res.

[11] Koshiyama M, Matsumura N, Konishi I (2016). Clinical efficacy of ovarian cancer screening. J Cancer.

[12] Mahmood S, Bhatti A, Syed NA, John P (2016). The microrna regulatory network: a far-reaching approach to the regulate the Wnt signaling pathway in number of diseases. J Recept Signal Transduct Res.

[13] Blyszczuk P, Muller-Edenborn B, Valenta T, Osto E, Stellato M, Behnke S, Glatz K, Basler K, Luscher TF, Distler O, Eriksson U, Kania G (2016). transforming growth factor-beta-dependent Wnt secretion controls myofibroblast formation and myocardial fibrosis progression in experimental autoimmune myocarditis. Eur Heart J.

[14] Giannakouros P, Comamala M, Matte I, Rancourt C, Piché A (2015). Muc16 mucin (Ca125) regulates the formation of multicellular aggregates by altering B-catenin signaling. Am J Cancer Res.

[15] Pradjatmo H (2016). Impact of preoperative serum levels of CA 125 on epithelial ovarian cancer survival. Asian Pac J Cancer Prev.

[16] van Altena AM, Kolwijck E, Spanjer MJ, Hendriks JC, Massuger LF, de Hullu JA (2010). Ca125 nadir concentration is an independent predictor of tumor recurrence in patients with ovarian cancer: a population-based study. Gynecol Oncol.

[17] Gupta D, Lammersfeld CA, Vashi PG, Braun DP (2010). Longitudinal monitoring of Ca125 levels provides additional information about survival in ovarian cancer. J Ovarian Res.

[18] Thibault B, Clement E, Zorza G, Meignan S, Delord JP, Couderc B, Bailly C, Narducci F, Vandenberghe I, Kruczynski A, Guilbaud N, Ferre P, Annereau JP (2016). F14512, a polyamine-vectorized inhibitor of topoisomerase ii, exhibits a marked anti-tumor activity in ovarian cancer. Cancer Lett.

[19] Galvan-Turner VB, Chang J, Ziogas A, Bristow RE (2015). Observed-to-expected ratio for adherence to treatment guidelines as a quality of care indicator for ovarian cancer. Gynecol Oncol.

[20] Lungchukiet P, Sun Y, Kasiappan R, Quarni W, Nicosia SV, Zhang X, Bai W (2015). suppression of epithelial ovarian cancer invasion into the omentum by 1alpha, 25-dihydroxyvitamin D3 and its receptor. J Steroid Biochem Mol Biol.

[21] Luo N, Guo J, Chen L, Yang W, Qu X, Cheng Z (2016). Arhgap10, downregulated in ovarian cancer, suppresses tumorigenicity of ovarian cancer cells. Cell Death Dis.

[22] Yan F, Wang X, Shao L, Ge M, Hu X (2015). Analysis of Uhrf1 expression in human ovarian cancer tissues and its regulation in cancer cell growth. Tumour Biol.

[23] Kim SH, Kim KY, Yu SN, Seo YK, Chun SS, Yu HS, Ahn SC (2016). Silibinin induces mitochondrial Nox4-mediated endoplasmic reticulum stress response and its subsequent apoptosis. BMC Cancer.

[24] Xing YN, Zhang JY, Xu HM (2016). The roles of serum Cxcl16 in circulating tregs and gastrointestinal stromal tumor cells. Onco Targets Ther.

[25] Carmosino M, Gerbino A, Schena G, Procino G, Miglionico R, Forleo C, Favale S, Svelto M (2016). The expression of lamin a mutant R321x leads to endoplasmic reticulum stress with aberrant Ca2+ handling. J Cell Mol Med.

