# Supplementary material for: The effect of CA125 on metastasis of ovarian cancer: old marker new function
Source: Oncotarget. 2017 Jun 7;8(30):50015–22. doi: 10.18632/oncotarget.18388 (PMC5564824; doi:10.18632/oncotarget.18388)
Supplement: Supplementary file 1 [file oncotarget-08-50015-s001.pdf]

The effect of CA125 on metastasis of ovarian cancer: old marker new function

SUPPLEMENTARY INFORMATION

Supplementary Table 1: The details material of clinical patients samples

See Supplementary File 1
