# Supplementary material for: The effect of CA125 on metastasis of ovarian cancer: old marker new function
Source: Oncotarget. 2017 Jun 7;8(30):50015–22. doi: 10.18632/oncotarget.18388 (PMC5564824; doi:10.18632/oncotarget.18388)
Supplement: Supplementary file 2 [file oncotarget-08-50015-s002.docx]

| **Supplementary Table 1: The details material of clinical patients samples** | | | | |
| --- | --- | --- | --- | --- |
| Number | Age | CA125 level(U/ml) | Metastasis | Metastasis Site |
|  |  |  | Y (+) N (-) |  |
| 1 | 51 | 202 | ﹣ | No |
| 2 | 40 | 51.5 | ﹣ | No |
| 3 | 68 | 81.5 | ﹣ | No |
| 4 | 80 | 84.3 | ﹢ | Omentum majus,Liver |
| 5 | 63 | 10.3 | ﹣ | No |
| 6 | 66 | 54.4 | ﹣ | No |
| 7 | 49 | 1193 | ﹣ | No |
| 8 | 39 | 76.6 | ﹢ | Right fallopian tube |
| 9 | 71 | 12.8 | ﹣ | No |
| 10 | 55 | 1584 | ﹢ | Omentum majus,Pelvic wall |
| 11 | 59 | 178.7 | ﹢ | Brain |
| 12 | 25 | 52.1 | ﹣ | No |
| 13 | 57 | 527.2 | ﹢ | Mediastinal lymph nodes |
| 14 | 56 | 55.4 | ﹣ | No |
| 15 | 59 | 2853 | ﹢ | Lymphonodus |
| 16 | 58 | 41.2 | ﹢ | Abdominal multiple |
| 17 | 65 | 730.9 | ﹢ | Anterior abdominal wall |
| 18 | 24 | 723.3 | ﹣ | No |
| 19 | 57 | 513.5 | ﹣ | No |
| 20 | 55 | 220.9 | ﹣ | No |
| 21 | 48 | 165.6 | ﹢ | Retroperitoneal |
| 22 | 53 | 153.8 | ﹢ | Peritonea |
| 23 | 52 | 77.9 | ﹣ | No |
| 24 | 58 | 519.2 | ﹣ | No |
| 25 | 66 | 956.5 | ﹣ | No |
| 26 | 39 | 2058 | ﹣ | No |
| 27 | 61 | 78.4 | ﹣ | No |
| 28 | 49 | 151.8 | ﹢ | Peritoneum,Omentum |
| 29 | 72 | 1558 | ﹢ | Omentum |
| 30 | 45 | 577.8 | ﹢ | Anterior rectal wall |
| 31 | 59 | 1095 | ﹢ | Omentum majus |
| 32 | 56 | 105.2 | ﹢ | Liver,Elvic cavity |
| 33 | 45 | 289.2 | ﹢ | Peritoneum,Omentum majus |
| 34 | 76 | 1523 | ﹣ | No |
| 35 | 50 | 71.4 | ﹣ | No |
| 36 | 50 | 71.4 | ﹢ | Omentum majus |
| 37 | 49 | 13.4 | ﹣ | No |
| 38 | 69 | 1189 | ﹣ | No |
| 39 | 57 | 5.6 | ﹢ | Omentum majus |
| 40 | 49 | 1638 | ﹢ | Omentum majus |
| 41 | 57 | 541.6 | ﹣ | No |
| 42 | 60 | 1737 | ﹣ | No |
| 43 | 33 | 1512 | ﹢ | Multiple retroperitoneal lymph nodes |
| 44 | 62 | 154.8 | ﹢ | Widespread metastasis |
| 45 | 60 | 147.9 | ﹢ | Omentum majus,Pelvic wall,Mesentery |
| 46 | 52 | 1004 | ﹢ | Omentum majus，Lymphonodus |
| 47 | 55 | 17.9 | ﹢ | Multiple retroperitoneal lymph nodes |
| 48 | 46 | 147.3 | ﹢ | Cervix uteri,Rectum |
| 49 | 52 | 6000 | ﹢ | Omentum majus,Peritoneum |
| 50 | 61 | 21269 | ﹢ | Multiple lymph nodes |
| 51 | 49 | 57.5 | ﹢ | Omentum majus,Right pelvic wall |
| 52 | 49 | 951.8 | ﹢ | Rectal wall |
| 53 | 28 | 510 | ﹢ | Omentum majus |
| 54 | 53 | 298.1 | ﹢ | Peritoneum，Omentum |
| 55 | 36 | 31.7 | ﹢ | Groin,Omentum majus |
| 56 | 60 | 301.3 | ﹢ | Pelvic cavity，Adrenal gland |
| 57 | 56 | 6000 | ﹢ | Peritoneum，Omentum,Lymph gland |
| 58 | 69 | 143.3 | ﹢ | Omentum majus，Pelvic floor |
| 59 | 61 | 632.8 | ﹢ | Omentum majus，Rectal wall |
| 60 | 51 | 1304 | ﹣ | No |
| 61 | 47 | 1169 | ﹢ | Omentum majus,Peritoneum |
| 62 | 61 | 91 | ﹢ | Fallopian tube,Pelvic floor,Peritoneum |
| 63 | 44 | 423.7 | ﹢ | Omentum majus,Peritoneum,Rectal wall |
| 64 | 70 | 1220 | ﹢ | Omentum majus,Peritoneum,Liver |
| 65 | 65 | 198.4 | ﹢ | Extensive abdominal metastasis |
| 66 | 62 | 594.7 | ﹢ | Widespread metastasis |
| 67 | 60 | 2752 | ﹢ | Omentum majus,Peritoneum |
| 68 | 74 | 1219 | ﹢ | Omentum majus,Peritoneum |
| 69 | 49 | 351.2 | ﹢ | Bladder,Omentum majus |
| 70 | 72 | 1172 | ﹢ | Omentum majus |
| 71 | 48 | 188.3 | ﹣ | No |
| 72 | 57 | 745.8 | ﹢ | Liver,Pelvic cavity |
| 73 | 59 | 113.4 | ﹢ | Pelvic cavity，Bladder wall |
| 74 | 61 | 378.6 | ﹣ | No |
| 75 | 23 | 28.4 | ﹣ | No |
| 76 | 42 | 285.8 | ﹢ | Pelvic wall |
| 77 | 49 | 74.6 | ﹣ | No |
| 78 | 51 | 61.3 | ﹣ | No |
| 79 | 53 | 392.8 | ﹢ | Omentum majus,Abdominal wall |
| 80 | 63 | 628.3 | ﹢ | Intraperitoneal,Pelvic cavity |
| 81 | 65 | 368.8 | ﹢ | pelvic wall,Peritoneum,Rectum |
| 82 | 25 | 47.9 | ﹣ | No |
| 83 | 44 | 6000 | ﹢ | Omentum majus |
| 84 | 43 | 1555 | ﹢ | Pleura,Omentum majus |
| 85 | 40 | 3.5 | ﹢ | Pelvic cavity,Retroperitoneal |
| 86 | 51 | 666.4 | ﹢ | Lymph |
| 87 | 31 | 15.1 | ﹣ | No |
| 88 | 57 | 20.2 | ﹣ | No |
| 89 | 38 | 168 | ﹣ | No |
| 90 | 76 | 6000 | ﹢ | Retroperitoneal lymph node |
| 91 | 60 | 23 | ﹢ | Extensive metastasis |
| 92 | 56 | 1355 | ﹣ | No |
| 93 | 57 | 638.8 | ﹣ | No |
| 94 | 54 | 194.8 | ﹣ | No |
| 95 | 49 | 187.9 | ﹣ | No |
| 96 | 36 | 1417 | ﹣ | No |
| 97 | 71 | 7.8 | ﹣ | No |
